# Disseminated *Magnusiomyces clavatus* (*Geotrichum clavatum*) infection following allogenic stem cell transplantation: a case report

**DOI:** 10.1128/asmcr.00045-24

**Published:** 2024-12-12

**Authors:** Rahmah S. Alzahrani, Donald C. Vinh, Ewa Rajda, Gizelle Popradi, Philippe J. Dufresne, Matthew P. Cheng

**Affiliations:** 1 Divisions of Infectious Diseases and Medical Microbiology, McGill University Health Centre, Montreal, Canada; 2 McGill University 5620 , Montreal, Canada; 3 Division of Hematology, McGill University Health Centre 54473 , Montreal, Canada; 4 Laboratoire de santé publique du Québec, Institut national de santé publique du Québec, Sainte-Anne-de-Bellevue, Canada; Vanderbilt University Medical Center, Nashville, Tennessee, USA

**Keywords:** *Magnusiomyces clavatus*, *Geotrichum clavatum*, *Saprochaete clavata*, infection, allogenic stem cell transplantation

## Abstract

**Background:**

*Magnusiomyces clavatus* (formerly *Geotrichum clavatum/Saprochaete clavata*) is a yeast with a filamentous form, as well as an uncommon, yet emerging opportunistic pathogen. It occurs primarily in patients experiencing prolonged neutropenia, and there is limited evidence available to guide antifungal agent selection and treatment decisions.

**Case Summary:**

We present a case of disseminated infection caused by *M. clavatus* in a patient with myelofibrosis following allogeneic hematopoietic stem cell transplantation. The patient was treated with voriconazole, as well as liposomal amphotericin B, along with granulocyte transfusion. The patient died from persistent fungemia, refractory septic shock, and multiorgan failure 20 days after the infection was first diagnosed.

**Conclusion:**

We subsequently discuss the epidemiological, clinical, and therapeutic features of this severe fungal infection and present a review of currently available literature.

## INTRODUCTION

Invasive fungal diseases (IFD) are life-threatening conditions most commonly observed in immunocompromised individuals, including those with malignancies or who receive immunosuppressive therapy, as well as solid organ or hematopoietic cell transplantation recipients (1). *Candida* and *Aspergillus* species are among the most prevalent agents responsible for IFD. However, other less common fungi are increasingly being recognized as causative agents of IFD and characterized by the international medical community (2). While *Magnusiomyces* is an infrequent genus associated with systemic disease, it has been previously described in immunocompromised individuals, such as those with hematological malignancies, with prolonged chemotherapy-induced neutropenia, or undergoing hematopoietic stem cell transplantation. Additionally, it has been observed in patients with diabetes, with solid organ transplantation history, or during their stay in the intensive care unit (3). Here, we present a case of disseminated infection caused by *Magnusiomyces clavatus* in a patient with allogeneic hematopoietic stem cell transplantation for myelofibrosis.

## CASE PRESENTATION

A 69-year-old man with myelofibrosis was electively admitted to a hospital in December 2023 for allogenic stem cell transplantation. The patient’s past medical history was significant for essential thrombocytosis diagnosed 3 years earlier and treated with hydroxyurea. One year prior to presentation, the patient developed transfusion-dependent anemia. For this reason, the patient underwent allogenic stem cell transplantation from a matched related donor. He received reduced intensity conditioning with fludarabine and busulfan, as well as low-dose total body irradiation. Antimicrobial prophylaxis consisted of oral trimethoprim/sulfamethoxazole, fluconazole, and valacyclovir started 1 day prior to stem cell transplantation. Prophylaxis against graft-versus-host disease consisted of tacrolimus and mycophenolate mofetil started 5 days post-transplant.

On the day of admission, a triple-lumen peripherally inserted central catheter (PICC) line was placed without complication. Three days prior to transplant, he developed one episode of fever to 38°C and received empiric intravenous piperacillin/tazobactam 3.375 g q6h. Blood cultures were negative, and he defervesced the next day but continued to receive intravenous piperacillin/tazobactam due to ongoing neutropenia. Twelve days post-transplantation, the patient developed a second fever to 39.5°C while receiving antimicrobial therapy. The treating team opted to modify empiric antimicrobial therapy and changed piperacillin/tazobactam to intravenous meropenem 1 g q8h and vancomycin 15 mg/kg q12h. Blood work revealed a white blood cell count of 0, transaminitis [asparate aminotransferase = 690 U/L (normal 8–33 U/L), alanine transaminase = 150 U/L (normal 4–36 U/L)] and hyperbilirubinemia [total bilirubin 65 µmol/L (normal 1.70–20 µmol/L)] at that time. The patient’s mental status was noted to decline as well.

The next day, the treating team consulted the infectious diseases service for a yeast isolated from blood cultures from both peripheral and PICC line cultures. The decision was made to continue the same empiric antibiotics, but to replace fluconazole with intravenous caspofungin 70 mg load on day 1, then 50 mg q24h; the patient’s PICC line was also promptly removed due to the presence of erythema around the catheter site. Transthoracic echocardiogram showed normal valves, and no vegetations were identified.

Fourteen days post-transplant, now 1 day after the yeast was isolated from the blood cultures, the patient suddenly developed increased oxygen requirements and worsening mental status. Laboratory investigations revealed an acute kidney injury [creatinine = 526 µmol/L (normal = 55–96 µmol/L)]. The patient was intubated, received intravenous crystalloid fluid resuscitation, and was started on multiple inotropic agents through a new central line.

**Fig 1:**
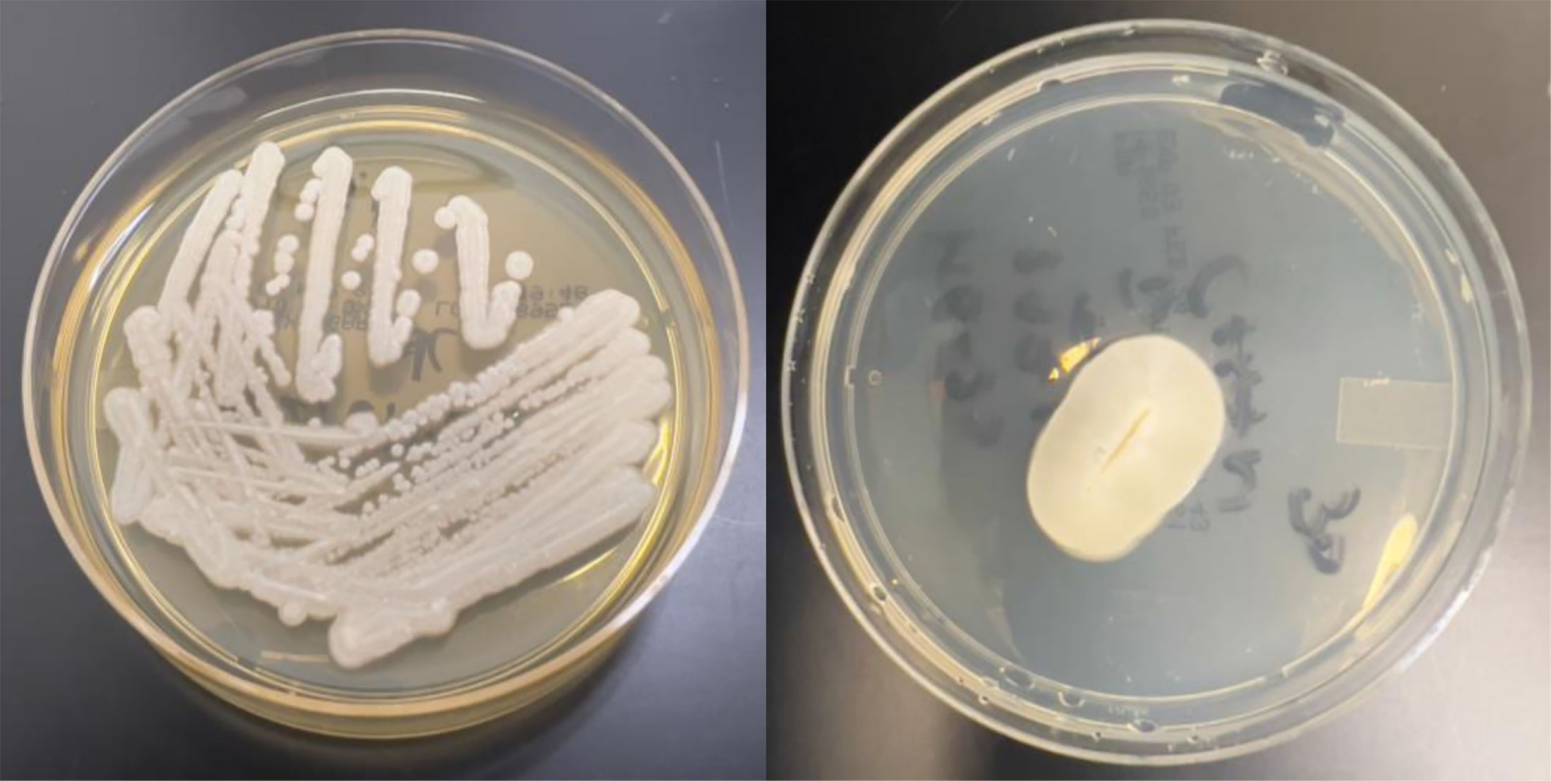
Macroscopic appearance of *Magnusiomyces clavatus* demonstrating white, dry, slightly cottony colonies with a frosted-glass appearance on Sabouraud agar after 7 days of incubation at 25°C.

**Fig 2:**
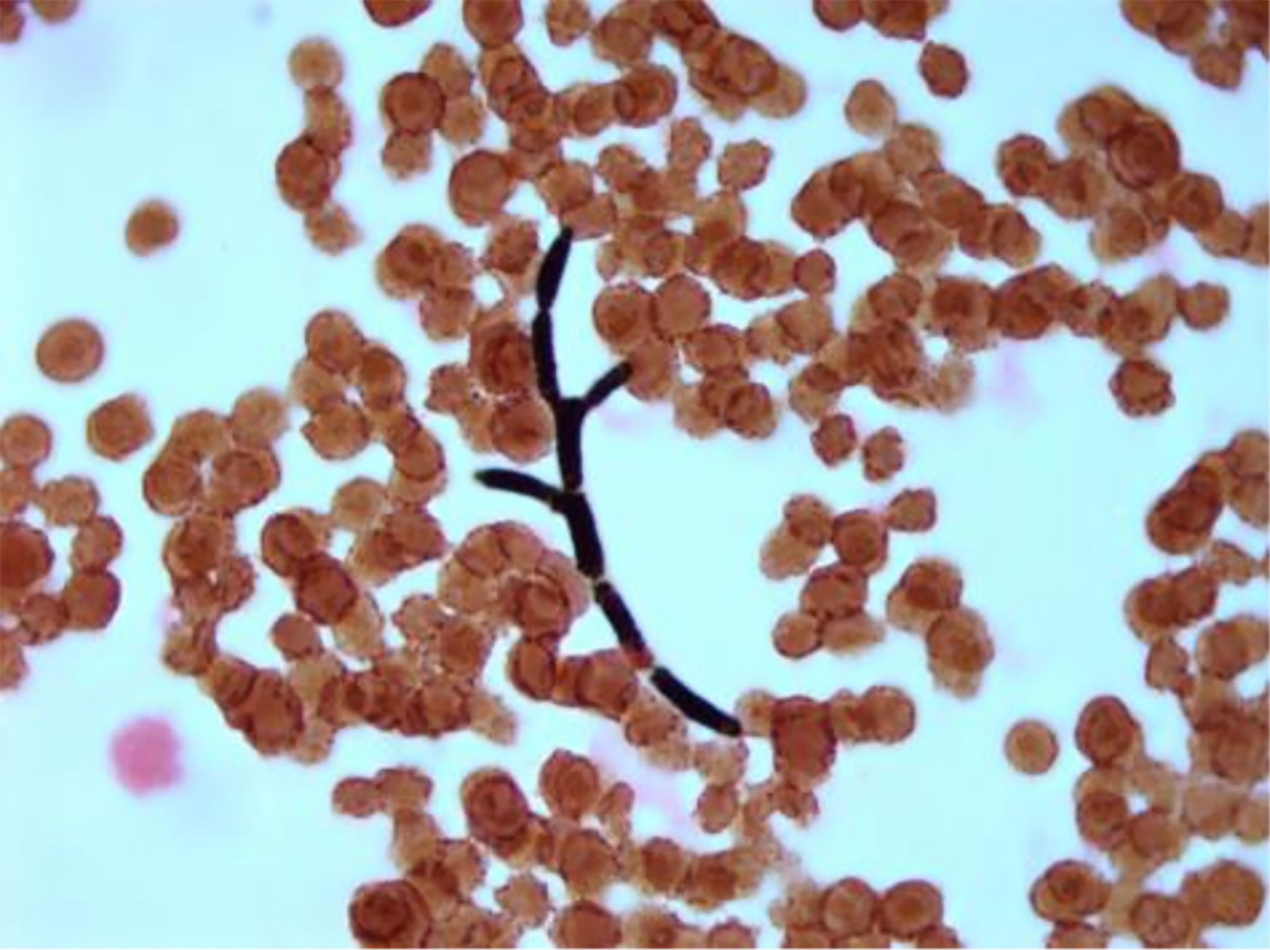
Gram stain preparation of the patient’s blood cultures showing septate hyphae.

**Fig 3:**
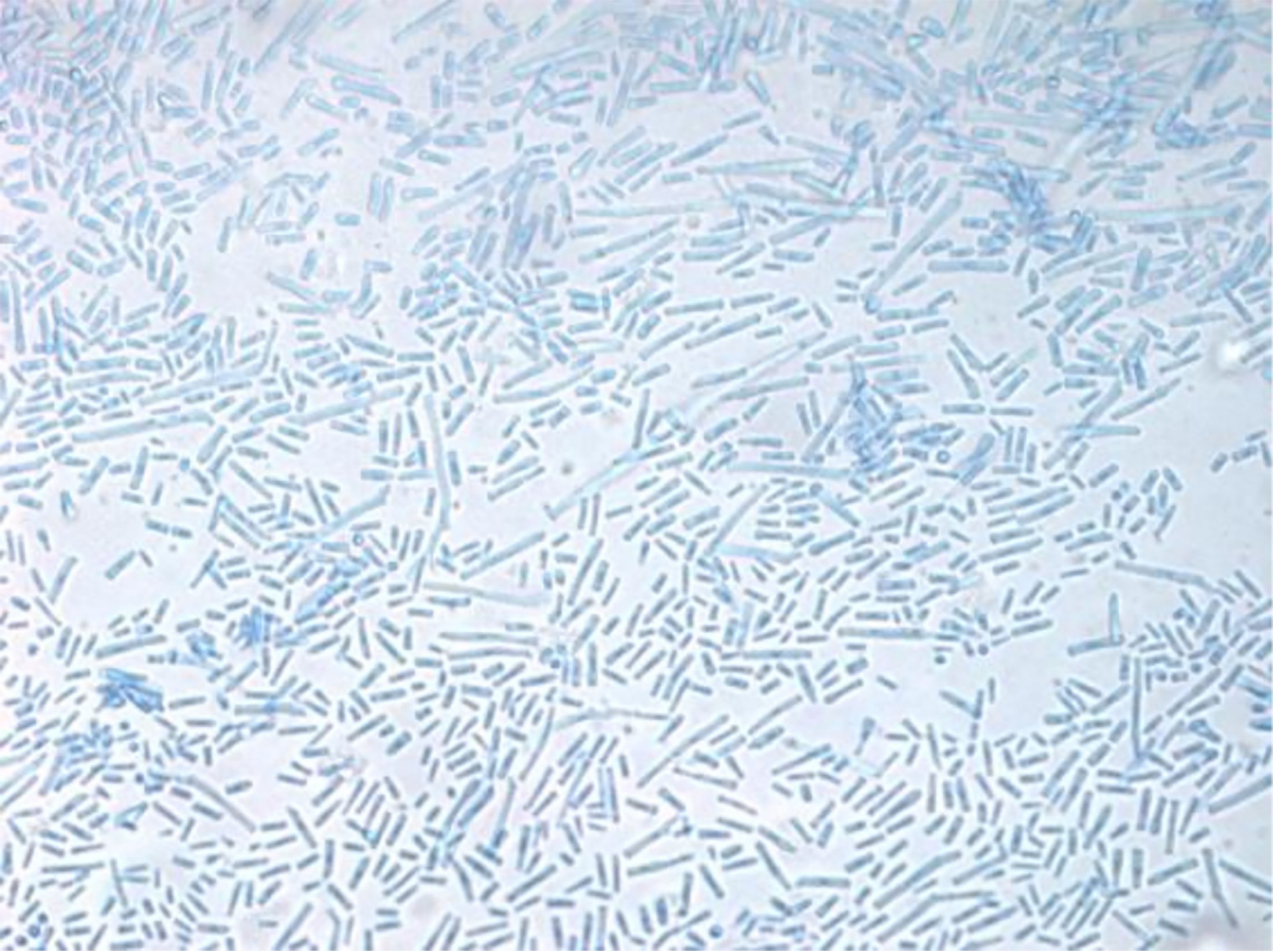
Lactophenol cotton blue image obtained by tape mount of *Magnusiomyces clavatus*, showing rectangular arthroconidia (magnification factor ×400).

The yeast from blood cultures grew on Sabouraud dextrose agar as white, dry, cottony colonies with a frosted-glass appearance (Fig. 1). Gram stain of blood culture demonstrated hyphal forms (Fig. 2). Microscopically, with lactophenol blue staining, it appears as hyaline septate hyphae segmented into chains of rectangular arthroconidia of quite variable size without producing blastoconidia (Fig. 3). The yeast was identified by matrix-assisted laser desorption ionization-time of flight mass spectrometry (MALDI-TOF MS) analysis on VITEK MS with V3.2 IVD database (bioMérieux) as *Geotrichum/Saprochaete* species with over 99% confidence. For this reason, intravenous voriconazole 6 mg/kg q12h and liposomal amphotericin B 5 mg/kg/day were started.

The fungal isolate’s identification was further confirmed by Laboratoire de santé publique du Québec (LSPQ) as *M. clavatus* (formerly *Saprochaete clavata*/*Geotrichum clavatum*) by ITS (primers ITS1 and ITS4) and D1D2 region (primers NL1 and NL4) sequencing. The definitive identification was obtained by local blast and full alignment to ISHAM ITS (https://its.mycologylab.org/), MycoBank (https://www.mycobank.org/Pairwise_alignment), GenBank nr databases https://www.ncbi.nlm.nih.gov/genbank/), and by direct alignment to *Magnusiomyces* and related species reference sequences. Accordingly, 100% and best sequence match was obtained against ex-type *M. clavatus* strain CBS 425.71 for both ITS (GenBank sequence KF984489.1) and D1D2 (GenBank sequence NG_055401.1) regions.

Antifungal susceptibility testing using CLSI broth microdilution methodology for yeasts (4) revealed low minimum inhibitory concentrations (MIC) to voriconazole, itraconazole, isavuconazole, posaconazole, and amphotericin B but high MIC to fluconazole and echinocandins (Table 1).

**TABLE 1:** Study of the *in vitro* antifungal susceptibility

Antifungal	MIC (mcg/mL)
Amphotericin B	1
Anidulafungin	4
Caspofungin	8
5-Fluorocytosine	≤2
Fluconazole	8
Itraconazole	0.25
Micafungin	1
Posaconazole	0.25
Voriconazole	0.12
Isavuconazole	0.25

The patient continued to have worsening mental status and multiorgan failure with persistent positive blood and urine fungal cultures despite exchanging central venous catheters while on antifungal therapy. The patient never exhibited neutrophil engraftment; thus, on 20-day post allo-SCT, because the patient remained critically ill, granulocyte infusions were initiated for 3 days. The patient had persistent fungemia and died 22 days post-transplant of refractory septic shock.

## DISCUSSION

We present a case of disseminated IFD caused by *M. clavatus* in a patient with myelofibrosis following allogeneic hematopoietic stem cell transplantation. Given the presence of erythema surrounding the catheter insertion site and blood cultures from the PICC line confirming the presence of *M. clavatus*, the central venous catheter was suspected to be the route of entry. We believe that the fungus rapidly disseminated hematogenously from that entry point, and the absence of a robust innate immune response in the setting of profound neutropenia was a determining factor in the patient’s demise.

From a diagnostic vantage point, a Gram stain from the blood sample can provide rapid and presumptive diagnosis of a fungemia. However, the culture of the organism remains critical for identification and diagnostic confirmation (5). While organism identification by MALDI-TOF MS is not always recommended for the identification of molds, it is generally very accurate for yeasts and correctly identified the species in our case (6), as confirmed by ITS and D1D2 sequencing.

Historically, *Geotrichum* was initially identified as *Trichosporon* spp. and grouped within the basidiomycota. Later, *Geotrichum* was reclassified as an ascomycete from a microbiological perspective primarily due to the presence of septal pores and its characteristic tendency to produce abundant arthroconidia and few blastoconidia. Recent advancements in microbiological techniques, particularly utilizing proteome and genome sequencing, have led to additional taxonomic classification of *Geotrichum*, with several new species of *Geotrichum* recently being described (7).

Around 96 cases of *M. clavatus* were reported in the literature. Several cases of rapidly fatal infections were reported in France in May 2012 within a brief timeframe across three healthcare facilities (8). Similar to our case, the majority of *M. clavatus* reported cases resulted in disseminated disease primarily affecting patients with underlying hematological malignancies with 60% mortality rate (9). A higher incidence of infection was observed among men and middle-aged individuals (9). In the majority of published cases, the source of infection remained unclear. However, potential sources included contaminated dairy products and implanted medical devises (3). Catheter-associated infection caused by *M. clavatus* has been reported in hematological malignances (10, 11), but only a few case reports on *M. clavatus* infection following allogenic stem cell transplantation have been described in the literature (9, 12). One case report described disseminated fungemia by *M. clavatus* 11 days post-stem cell transplant with cutaneous involvement and possible brain, liver, and lung involvement similar to our case with poor outcome with the combination therapy of liposomal amphotericin B and 5-fluorocytosine (13). In our case, the potential for rapid involvement of multiple organs highlights the challenges faced in managing such an aggressive infection caused by *M. clavatus*.

In our case, *in vitro* data indicated relatively low MIC to voriconazole, itraconazole, isavuconazole, posaconazole, and amphotericin B and higher MIC to fluconazole and the echinocandins (Table 1). This is consistent with the *Magnusiomyces* species susceptibility profile found in the literature (3). While no established breakpoints or epidemiological cut-off values (ECV) exist for interpreting the MICs of various antifungals against *M. clavatus,* recent guidelines (5) would recommend avoiding echinocandins due to concerns of intrinsic resistance. Despite the MIC value of 1 mcg/mL to micafungin, we opted against its use for definitive therapy due to the range of MICs reported for other echinocandins and the aforementioned concern of antifungal resistance (5). The limited number of reported cases of *M. clavatus* infections does not allow the ability to define the optimal antifungal treatment. In cases with severe or invasive *Magnusiomyces* infection, the available literature and recent guidelines (5) support the use of liposomal amphotericin B with or without flucytosine, with limited data regarding the efficacy of voriconazole or combination of antifungal agents (14, 15). For this reason, and considering the remarkably low MIC to voriconazole (0.12 mcg/mL), we opted to pursue definitive antifungal therapy with both liposomal amphotericin B and voriconazole.

A single reported case of disseminated *M. clavatus* invasive infection in a patient with aplastic anemia demonstrated successful treatment with a combination of voriconazole, liposomal amphotericin B, and adjunctive granulocyte transfusions (12). Combination therapy with caspofungin and voriconazole with potential *in vitro* synergy or voriconazole and liposomal amphotericin B has also been reported and provided mixed successes, and the use of combination therapy remains controversial (16).

In the case we described, we elected to empirically add liposomal amphotericin B to voriconazole as the patient’s clinical condition rapidly deteriorated. In our patient, the fungemia persisted despite amphotericin B and voriconazole therapy and supportive measures like granulocyte transfusion. This highlights the importance of addressing potential sources of the infection, such as removing the endovascular lines, and reversing any underlying immunosuppression, if possible.

### Conclusion

*M. clavatus* is an emerging cause of invasive fungal diseases, posing a significant threat to profoundly immunosuppressed patients. It is crucial to consider this organism in the differential diagnosis of disseminated fungal infections in immunocompromised patients, especially when not responding to echinocandins. Rapid identification via MALDI-TOF and performing susceptibility testing are essential in guiding the selection of appropriate antifungal therapy. Additionally, further understanding of rare causative agents of IFD is crucial for improving treatment outcomes in affected patients.
